# Using item response theory to explore the psychometric properties of extended matching questions examination in undergraduate medical education 

**DOI:** 10.1186/1472-6920-5-9

**Published:** 2005-03-07

**Authors:** Bipin Bhakta, Alan Tennant, Mike Horton, Gemma Lawton, David Andrich

**Affiliations:** 1 Academic Unit of Musculoskeletal and Rehabilitation Medicine, University of Leeds, UK; 2 School of Education, Murdoch University, Western Australia

## Abstract

**Background:**

As assessment has been shown to direct learning, it is critical that the examinations developed to test clinical competence in medical undergraduates are valid and reliable. The use of extended matching questions (EMQ) has been advocated to overcome some of the criticisms of using multiple-choice questions to test factual and applied knowledge.

**Methods:**

We analysed the results from the Extended Matching Questions Examination taken by 4^th ^year undergraduate medical students in the academic year 2001 to 2002. Rasch analysis was used to examine whether the set of questions used in the examination mapped on to a unidimensional scale, the degree of difficulty of questions within and between the various medical and surgical specialties and the pattern of responses within individual questions to assess the impact of the distractor options.

**Results:**

Analysis of a subset of items and of the full examination demonstrated internal construct validity and the absence of bias on the majority of questions. Three main patterns of response selection were identified.

**Conclusion:**

Modern psychometric methods based upon the work of Rasch provide a useful approach to the calibration and analysis of EMQ undergraduate medical assessments. The approach allows for a formal test of the unidimensionality of the questions and thus the validity of the summed score. Given the metric calibration which follows fit to the model, it also allows for the establishment of items banks to facilitate continuity and equity in exam standards.

## Background

It is acknowledged from medical student learning behaviour that assessment often drives learning [1]. Therefore, if students are learning what is being assessed then it is vital that the content of the assessment reflects the learning objectives. This process, known as blueprinting, maps the content of assessments against the clinical competencies (knowledge, skills and attitudes) that the student is expected to acquire [2]. The pyramid of competence developed by Miller provides a conceptual framework for ensuring that student assessments are valid and cover core aspects of factual knowledge and problem solving (e.g. Extended Matching Questions – EMQ), performance assessment in "vitro" (e.g. Objective Structured Clinical Examinations – OSCE) and performance in "vivo" (e.g. case presentations, log books) [3].

At the University of Leeds the undergraduate medical course includes an integrated medical and surgical specialities program (Rheumatology, Orthopaedics, Rehabilitation, Anaesthetics, Dermatology, Infectious diseases, Oncology and Palliative Medicine, Genitourinary Medicine and Accident and Emergency medicine). Acknowledging that no single assessment format can adequately assess all the learning objectives within the course blueprint, a combination of assessments (including OSCE, EMQ, slides with problem solving, reflective learning log books and case presentations) are currently used to assess the student's competence. Although a combined score is used to assess the competence of the students, test scores within the individual assessments reflect a richer profile of the individual, allowing an understanding of strengths and weaknesses that can result in improvement in the individual and the educational programme. Analysis of the quality of individual assessments is essential to this process. The focus of this paper is the use of item response theory to examine the validity of the typical use of a single score obtained from the summative EMQ examination, to characterise each student and their individual differences, in short the investigation of the relative unidimensionality of the EMQ examination

**Figure 1:**
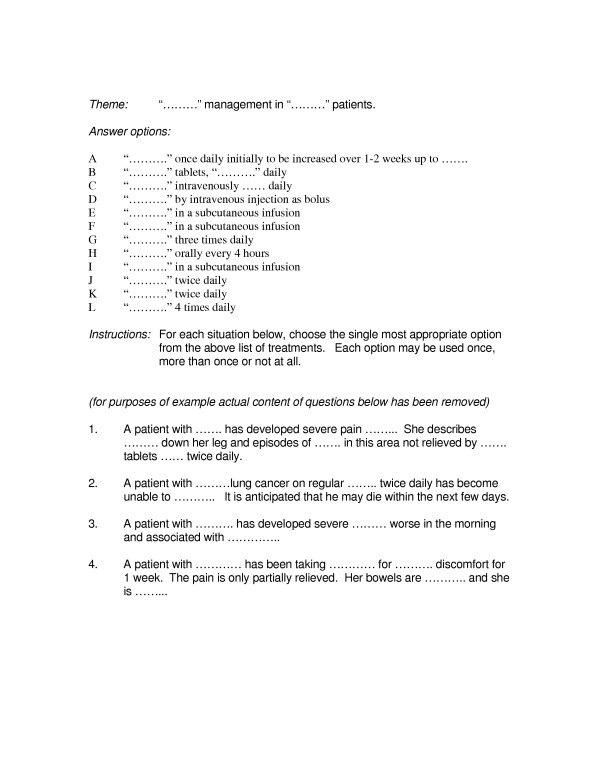
Example of Extended Matching Question (EMQ) Format.

The EMQ is a form of multiple-choice type question [4] designed to test the student's knowledge. EMQs are written by experts from each of the medical specialties. EMQs have four components; a *theme *(e.g. leg or cancer pain), the *lead-in *for the questions that gives the students instructions on what to do (e.g. "for each patient select the most likely diagnosis"); the *questions *in the form of vignettes giving the pertinent information based on which the student is to select the correct answer; and finally the potential *answers *(e.g. a list of potential diagnoses relevant to the theme) (Figure 1). The response option includes one correct answer for each question, and other possible responses as distractors, a reasonably plausible response if the student does not know the correct response for whatever reason.

The use of EMQs has been advocated to overcome some of the criticisms levelled at the use of multiple-choice questions to test factual and applied knowledge. There are advantages to using EMQs [4]:

• The format of themes aid the organisation of the examination, and the use of blueprinting is a natural aid to the process of writing EMQs

• As questions are written in themes or general topic it allows the teacher to write many questions for that theme and then share these questions out randomly to create more than one examination paper

• Good questions provide a structure designed to assess application of knowledge rather than purely recall of isolated facts

• The approach to writing these questions is systematic, which is very important when several people are contributing questions to one exam

• The extended list of options allows the inclusion of all relevant options, and reduces the opportunity for students to 'guess' the correct answer as in MCQs

• EMQs were found to be more discriminating than two and five option versions of the same questions resulting in a greater spread of scores, and reliability was higher as a consequence of this [5,6].

The importance of ensuring validity and reliability of the EMQ procedure is crucial. Current evidence for this is limited but does support good reliability and validity [1,6,7]. This study considers EMQs as used in the medical education at two levels. Within the individual EMQ it explores the operation of the distractors, and across EMQs it explores whether (a) summary scores are justified; (b) how EMQs vary in difficulty across specialities and (c) whether the EMQ scores taken together discriminate students of different abilities.

Traditionally, methods of analysis based on classical test theory have been used to evaluate such tests. The focus of the analysis is on the total test score; frequency of correct responses (to indicate question difficulty); frequency of responses (to examine distractors); reliability of the test and item-total correlation (to evaluate discrimination at the item level) [8-11]. Although these statistics have been widely used, one limitation is that they relate to the sample under scrutiny and thus all the statistics that describe items and questions are sample dependent [12]. This critique may not be particularly relevant where successive samples are reasonably representative and do not vary across time, but this will need to be confirmed and complex strategies have been proposed to overcome this limitation.

Developments of the Classical Test Theory can be found in modern test theory and, in particular, the Rasch model [13] of Item Response Theory (IRT). This too uses the total test score, but in this instance from a theoretical basis. It is also concerned with reliability (in the form of a person separation statistic which is similar to Cronbach's alpha). However, in addition it provides a mechanism for testing the invariance of items which allows the construction of a bank of calibrated questions that facilitates a direct comparison over different administrations of the test [14]. From such an item bank, different combinations of questions can be incorporated into an examination to ensure that the difficulty of the exam remains consistent for successive cohorts of students.

The use of the Rasch model entails a different perspective, or paradigm, from IRT approaches in general [15]. Where data do not conform to the expectations of the Rasch model, the main challenge is *not *to find a model that better accounts for the data, but to understand statistical misfit as *substantive anomalies *that need to be understood, and by being understood, to lead to the construction of more valid and reliable tests. This is the approach taken in this study. That is, analysis of data based on existing items will be considered closely both statistically and substantively with a view to guiding better question construction. Thus the aim of this paper is to explore the use of Rasch analysis to determine the validity of the EMQ examination currently taken by 4^th ^year medical undergraduates.

## Methods

### Data collection

Responses to the EMQ examination taken by one hundred and ninety three 4^th ^year medical students were used as the source data. The examination is designed to test factual and applied knowledge taught in the Medical and Surgical Specialties course and is taken by the students at the end of the 16 weeks course. The course is run three times per year and rotates with two other courses (Paediatrics / Obstetrics / Gynaecology *and *Primary Care / Public Health / Psychiatry). All questions were devised by the lead educational supervisor within each specialty. Training in EMQ writing was provided to the medical specialty supervisors. The examination consisted of 98 EMQs distributed across eight specialties and 27 themes, each with two to four EMQs. Each themed group of EMQs had eight to 15 possible response options (e.g. see example in Figure 1). There were 12 Oncology, 14 Anaesthetics, 12 Dermatology, 12 A&E, 12 Infectious Diseases, 16 Orthopaedics, 8 Rheumatology and 12 Rehabilitation EMQs. The final exam mark is the sum of correct answers to all themes, summed across specialties, giving an indication of the applied knowledge across the range of medical and surgical specialties which comprised the Medical and Surgical Specialities module.

No other information was collected about the students other than which term they had sat the EMQ examination. The students take this examination at the end of the course and the medical and surgical specialties course is repeated three times a year. Differential Item Functioning (see below) was used to determine the impact of the term in which the examination was taken on student performance.

### Parameter estimation

The Rasch model is a probabilistic unidimensional model which asserts that (1) the easier the question the more likely the student will respond correctly to it, and (2) the more able the student, the more likely he/she will pass the question compared to a less able student. The model assumes that the probability that a student will correctly answer a question is a logistic function of the *difference *between the student's ability [*θ*] and the difficulty of the question [*β*] (i.e. the ability required to answer the question correctly), and only a function of that difference



From this, the expected pattern of responses to questions can be determined given the estimated *θ *and *β*. Even though each response to each question must depend upon the students' ability and the questions' difficulty, in the data analysis, it is possible to condition out or eliminate the student's abilities (by taking all students at the same score level) in order to estimate the relative question difficulties [14,16]. Thus, when data fit the model, the relative difficulties of the questions are independent of the relative abilities of the students, and vice versa [17]. The further consequence of this invariance is that it justifies the use of the total score [18,19]. In the current analysis this estimation is done through a pair-wise conditional maximum likelihood algorithm, which underlies the RUMM2020 Rasch measurement software [20,21]

**Figure 2:**
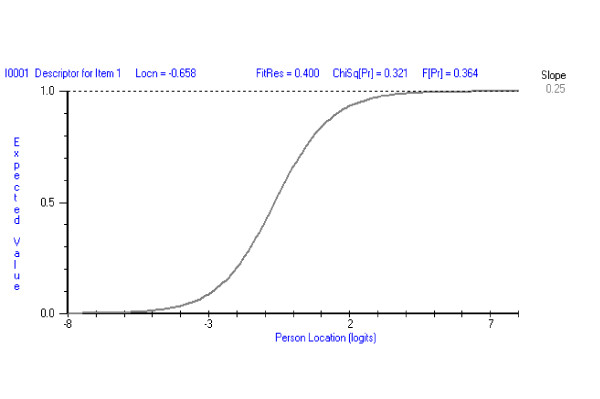
An Item Response Function (Item Characteristic Curve).

If the above assumptions hold true then the relationship between the performance of students on an individual question and the underlying trait (applied knowledge within the medical and surgical specialties course) can be described by an S shaped curve (item response function). Thus the probability of answering the question correctly consistently increases as the location on the trait (knowledge) increases (Figure 2). The steepness of the curve indicates the rapidity with which the probability that a student responding to the question correctly, changes as a function of this location (ability). The location of the curve along the horizontal axis (defined by the point at which the 0.5 probability level bisects the horizontal scale) indicates the difficulty of the question. The location of the student on the same axis indicates their level (of knowledge, ability etc.) on the trait.

When the observed response pattern does not deviate significantly from the expected response pattern then the questions constitute a true measurement or Rasch scale [22]. Taken with confirmation of local independence of questions, that is, no residual associations in the data after the person ability (first factor) has been removed, this supports the unidimensionality of the scale [23,24].

### General tests of fit

In this analysis, responses to the EMQ are analysed as dichotomous options, that is, one correct answer and all of the other options are analysed together as one incorrect response. To determine how well each question fits the model, and so contributes to a single trait, a set of 'fit' statistics are used which test how far the observed data match those expected by the model. The trait refers to the required knowledge base that the student must acquire within the medical and surgical specialties course. The Item – Trait Interaction Statistic (denoted by the chi-square value), reflects the degree of invariance across the trait. A significant chi-square value indicates that the relative location of the question difficulty is not constant across the trait. In addition, question fit statistics are examined as residuals (a summation of the deviations of individual students responses from the expected response for the question). An estimate of the internal consistency reliability of the examination is based on the Person Separation Index where the estimates on the logit scale for each person are used to calculate reliability.

Misfit of a question indicates a lack of the expected probabilistic relationship between the question and other questions in the examination. This may indicate that the question does not contribute to the trait under consideration. In the current study students are divided into three ability groups (upper third, middle third and lower third) denoting each Class interval with approximately 65 students in each. Furthermore, significance levels of fit to the model are adjusted to take account of multiple testing (e.g. for 24 items the level would be 0.002 and for 98 the level would be 0.0005) [25].

As well as invariance across the trait, questions should display the same relative difficulty, irrespective of which externally defined group is being assessed. Thus, the probability of correctly answering a question should be the same between groups given the same ability level of the student. For example, given the same ability, the students should not be more likely to answer a question correctly simply because they sat the exam in the third term instead of the first or second term. This type of analysis is called Differential Item Functioning (DIF) [26]. The basis of DIF analysis lies in the item response function, and the proportion of students at the same ability level who correctly answer the question. If the question measures the same ability across groups of students then, except for random variations, the same response curve is found irrespective of the group for whom the function is plotted [26]. Thus DIF refers to questions that do not yield the same response function for two or more groups (e.g. gender or the cohort of students).

DIF is identified by two way analysis of variance (ANOVA) of the residuals with the term in which the examination was taken by the student as one factor and the class interval as the other [27]. Two types of DIF are identified: (a) uniform DIF demonstrating that the effect of the term in which the exam was taken are the same across all class intervals (main effect), and (b) non-uniform DIF which demonstrates that the effect of which term the student sat the exam in is different across class intervals (interaction effect). Where there are more than two levels of a factor, Tukey's post hoc test is used to indicate which groups are contributing to the significant difference.

Although EMQ are analysed as though they have dichotomous response categories (correct or incorrect), it is possible to examine how the separate incorrect options within an individual EMQ are contributing to the student's response. This procedure is very similar to the technique of Graphical Item Analysis(GIA) [28], though in this case the RUMM 2020 programme [21] produces the analysis with no extra user effort. The proportions of students in each class interval who have selected the various response categories, including the correct option, are plotted on a graph of the item response function. This visually illustrates how often the various response options are being selected by the students in relation to one and other, and can be compared across themes given that different options are likely to have different response patterns for different questions within a theme. This is particularly useful in improving the quality of the distractor responses.

In view of our limited sample size (and particularly the ratio of students to items) we elected in the first instance to examine in detail the psychometric properties of the musculoskeletal component of the EMQ examination, acknowledging the limitations associated with the accuracy of the person estimate based upon 24 items (29). Subsequent analysis of the whole examination is reported to demonstrate the potential benefits of using Rasch analysis, but again acknowledging the limited conclusions that can be drawn on student ability and question difficulty estimates for the whole examination as a result of looking at 98 items with 193 students.

## Results

Data were collected from 193 students (Term 1 = 61, Term 2 = 64, and Term 3 = 68). Total scores ranged from 36 to 78 out of a maximum mark of 98 (mean = 60.3, median = 61). Initially, analysis of data was undertaken from the combined specialties of rheumatology and orthopaedic questions, which consisted of 24 EMQs'.

### Analysis of the musculoskeletal component of the EMQ examination

To estimate individual question difficulty using the Rasch model, all the incorrect response options were treated together as one incorrect option. The fit of the 24 questions to the Rasch model was acceptable, both in terms of individual item fit (Table 1) and over all Item-Trait Interaction (χ^2 ^= 79.73, p = 0.003). This suggests that the musculoskeletal questions mapped on to a single dimension of applied knowledge in this case and within the power of the test of fit. This was further supported by a principal components analysis of the residuals identifying a first residual factor accounting for just 8% of the variation. However, the Person Separation Index was low, 0.50, indicating a low reliability. This, however, can be ascribed to the intrinsic homogeneity of the students who are selected under rigorous criteria and who are all studying for the same exam.

**Table 1:** Individual Item difficulty (location) and Tests of Fit (residuals and chi-square and its probability) for the 25 musculoskeletal EMQs.

Question	Location	SE	Residual	ChiSq	DF	Prob
OR63	-2.68	0.47	-0.60	0.06	1	0.81
OR64	-2.32	0.40	-0.67	0.43	1	0.51
OR65	-2.03	0.35	-1.02	0.96	1	0.33
OR66	1.03	0.15	2.48	1.85	1	0.17
OR67	-2.27	0.39	-1.12	2.32	1	0.13
OR68	1.74	0.16	0.20	0.02	1	0.88
OR69	-0.29	0.19	-0.79	0.52	1	0.47
OR70	-3.49	0.68	-0.25	0.59	1	0.44
OR71	0.81	0.16	1.62	1.19	1	0.28
OR72	-1.66	0.30	-0.52	0.44	1	0.51
OR73	-0.48	0.20	0.21	0.47	1	0.49
OR74	1.79	0.16	1.64	7.80	1	0.01
OR75	0.12	0.17	-0.64	0.33	1	0.57
OR76	1.70	0.16	-0.71	3.90	1	0.05
OR77	1.12	0.15	-0.52	2.95	1	0.09
OR78	1.20	0.15	1.76	0.08	1	0.78
RH79	-1.47	0.28	-0.61	1.00	1	0.32
RH80	1.57	0.15	-1.00	2.01	1	0.16
RH81	0.22	0.17	1.52	4.22	1	0.04
RH82	1.11	0.15	1.01	0.00	1	0.96
RH83	-1.08	0.24	-0.58	0.43	1	0.51
RH84	2.08	0.16	-0.18	0.09	1	0.76
RH85	1.50	0.15	-0.12	0.00	1	0.99
RH86	1.78	0.16	-0.88	3.28	1	0.07

OR** representing Orthopaedic EMQ

RH** representing Rheumatology EMQ

ChiSq Chi – squared statistic

SE Standard error

Location Value identifies question difficulty on logit scale

Residual Fit of question to underlying trait

DF Degrees of freedom

None of the items displayed DIF by the term in which the examination was taken. The *location *(a logit scale – Table 1) measures the item difficulty of the musculoskeletal EMQs and shows the range of difficulties from easy to hard (negative values indicating easier questions and positive values indicating harder questions).

### Analysis of the whole EMQ examination (all eight specialties)

**Figure 3:**
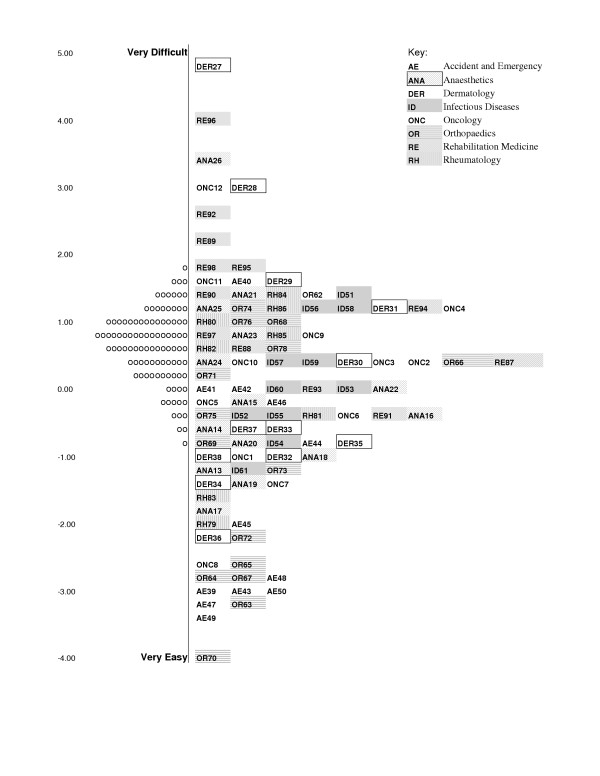
Map of question difficulty and student ability on Rasch transformed logit scale. Right hand side shows questions in order of difficulty and the left hand side shows the distribution of the students abilities based on their total examination score.

An initial exploration of the overall fit of the 98 questions to the Rasch model was poor with a significant question – trait interaction (χ^2 ^= 291.4, p < 0.0001). Three out of the 98 EMQs showed significant misfit to the model at the individual level; one Infectious Diseases question, one Oncology question and one Rehabilitation Medicine question. Once these misfitting questions were omitted from the analysis, the overall fit of the remaining 95 EMQs to the Rasch model improved, and showed no significant Item – Trait Interaction. The questions from each of the component specialties within the course had reasonable spread of difficulty across the logit scale (Figure 3). Overall the students were more likely to answer the A&E EMQs correctly than other themes. Five questions (Oncology2, Oncology3, A&E41, Rheumatology84 and Rehabilitation93) displayed DIF by term in which the examination was taken (p < 0.01) indicating that the student's responses to these questions were influenced by the term in which the students sat the examination. Note that when the overall trait from all specialties is considered, Rheumatology84 shows DIF, but not when just the musculoskeletal-related trait was considered.

### Response option analysis

The analysis of response options is descriptive. The proportions of students who have selected the various response categories, including the correct option, are plotted on a graph of the item response function. This visually illustrates student behaviour in their response to questions.

Three main patterns of response to the questions were identified.

#### a) the incorrect response option selected more frequently than the correct answer

**Figure 4:**
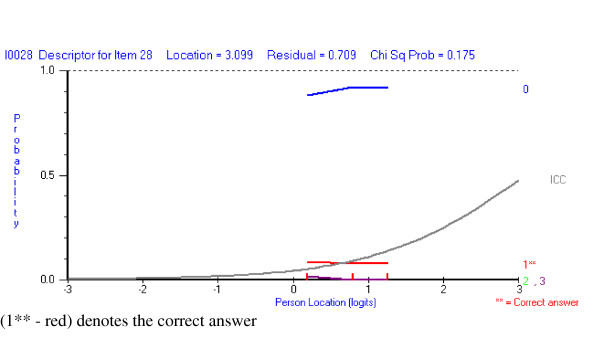
A question where the vast majority of the students are selecting the same incorrect response option (0 – blue).

The *majority *of the students are selecting the same wrong answer, irrespective of their ability level (Figure 4). In contrast, a more typical pattern of responses to a hard question would be students choosing a range of incorrect response options randomly, rather a single option.

#### b) the distractor response option is selected more frequently than right answer in some ability groups

**Figure 5:**
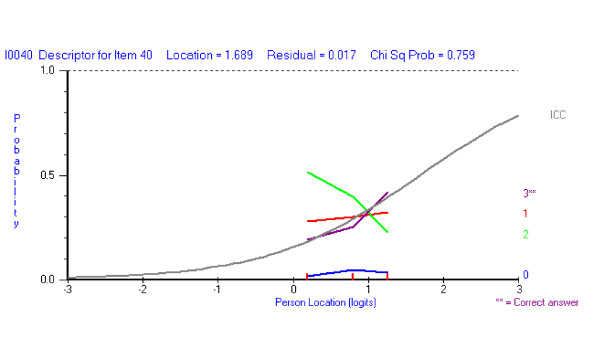
Distractor response option (2 – green) selected more often than the correct answer (3**) by lower ability students, but not higher ability students.

Responses to this EMQ show that students with lower ability are likely to select the incorrect answer, while the more able students select the correct response (Figure 5). Response option 0 is considered by most to be wrong, but options 1 and 2 clearly attract students of lesser ability.

#### c) the correct answer too obvious

**Figure 6:**
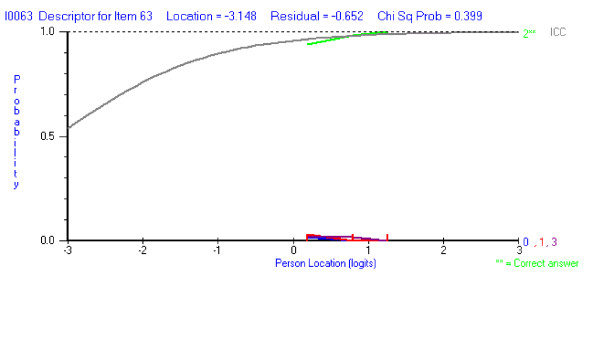
Correct answer is too obvious (2** – green).

The majority of the students in all ability groups select the correct answer (Figure 6). An "easy" question is not in itself undesirable as it may test the student on a critical piece of knowledge.

## Discussion

In this study, Rasch methodology was used to analyse the Extended Matching Questions examination taken by our fourth year medical students in 2001. Analysis of the musculoskeletal subset of questions showed that they mapped on to the trait under investigation (assessment of the musculoskeletal applied knowledge content of the course) and thus supported the notion of a valid unidimensional scale. Exploratory analysis of the whole examination showed that only three out of the 98 EMQs displayed significant misfit to the measurement model. These EMQs should be reviewed for ambiguities in question format and relationship to the course blueprint. DIF was identified in five of the 98 questions (these were different to the items displaying misfit) suggesting that the pattern of student responses to these EMQs were dictated by the term in which the exam was undertaken. The reason for this apparent variation needs to be understood before such questions are included into the bank of EMQs used for future examinations. In the present analysis only 'term' was considered for DIF, but DIF by gender also needs to be considered as gender can influence the response students make to the distractors [30].

Rasch analysis revealed the variety of response patterns made by the students. In some questions *the incorrect response is selected more frequently than correct response*, suggesting that the question appears extremely hard in that the majority of students are consistently selecting the same wrong option. This may relate to the stem being ambiguous, poor delivery of the teaching with students having experienced difficulty in understanding the topic such that the chosen response seems plausible to everyone.

In those questions where *the incorrect response is selected more frequently than the correct response but in only some ability groups *(distractor), this suggests that the distractor option creates a strongly discriminating question. In this case less able students are more likely to select a distractor response while the more able students are likely to select the correct option.

Where *most students select the correct answer, regardless of their overall ability level*, the question provides little information to distinguish the ability of the students. Kehoe [31] has suggested that "questions that virtually everyone gets right are useless for discriminating between students and should be replaced by more difficult items", particularly in the case where the pass mark of the exam is criterion referenced. However as the question might test an essential component of the course that is important for all students to know it may be reasonable for such questions to be included in the examination even though they may be poor discriminators of low and high ability students. The content of the examination needs to have a mixture of questions that are discriminatory as well as those that define the pass standard (which would include questions that appear to be too easy but test an essential component of the course). If a question is too easy and does not test an essential component of the course then it needs to be revised.

The data presented in this paper on the analysis of response pattern within individual questions is purely descriptive. Although visual inspection of the response patterns is informative, Wang [32] is currently developing a quantitative Rasch model based on the analytical approach traditionally used in Classical Test Theory. This alternative would appear to provide a more statistical, as opposed to descriptive analysis about how the response options are working within the context of the Rasch measurement model.

The use of the extended matching format with up to 14 response options reduces the impact of guessing on the student's overall mark compared with standard multiple choice questions. However, it could also be argued that setting questions within themes puts the test at risk of a breach of the local independence assumption, in that responses to questions on the same theme may be locally dependent. The PCA of the residuals reject this and support the assumption of local independence.

Rasch analysis also allows for the development of a bank of questions that have been calibrated with one another in terms of their difficulty. This allows different examinations to be constructed (from combinations of the calibrated questions) while retaining the same level of overall difficulty. This will reduce the likelihood of students in consecutive years taking harder or easier exams when the standard they have to attain is unchanged.

**Figure 7:**
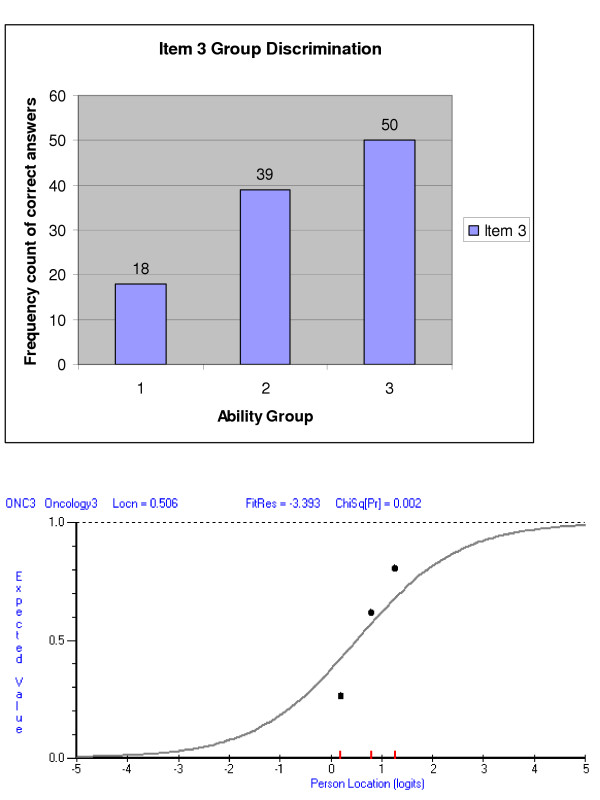
Graph of a misfitting EMQ (classic test theory and Rasch); EMQ 3.

Classical test theories (including Generalisability theory), widely used to investigate the quality of student assessment, make few assumptions about the characteristics of the questions such as whether they form a unidimensional construct. Therefore this approach can be used in a variety of measurement situations. A comparison between the classical approach and the Rasch approach with regard to discrimination is given in Figure 7. However the statistics obtained from classical analysis only apply to the specific group of students who took the test (i.e. the analysis is sample dependent). This analysis cannot separate the attributes of the questions from the attributes of student (e.g. ability) making it difficult to compare the performance of different sets of students who take the same format examinations with year on year content variations.

In contrast, the Rasch measurement model checks two important assumptions: (a) the probability of answering one question correctly does not increase the probability of answering another question correctly within the examination (local independence) and (b) all questions in the examination map on to one construct (unidimensionality). With respect to a) above, questions that incorporate sections whose answers influence the response to other sections within the same question cannot be analysed using this approach and, to b), unidimensionality is a requirement for the summation of any set of items (33).

In addition, arguments have been made to use the two parameter and three parameter logistic models (the latter which adds a guessing parameter) [34] as these much better reflect the type of curve derived from educational data. Unfortunately, apart from sample size requirements which are very high for these type of model, it is known that almost 100% of the time their parameters violate interval scaling [35]. Thus these models do not provide the invariance or quality of measurement which is required for summative unidimensional scales. The property of the invariance of the ratio of difficulty across items (that is this ratio between any two items is constant, irrespective of the ability of the students) is again an essential requirement for measurement.

Finally, a consistent problem with criterion related tests is the apparent low reliability, as expressed by a low person separation index. This is to be expected, as traditional tests of reliability are not appropriate for criterion-referenced tests [36] where the score distribution is likely to be very narrow.

## Conclusion

Case and Swanson [4,5] have set out clear guidelines on how to write an EMQ. The Rasch measurement model, and the associated analysis used in this study, will ideally be the next stage in the process of EMQ writing. It can be used to give feedback to the question writers on how to revise the problem questions. The analysis clearly shows how students use options, and this information coupled with expert knowledge and understanding of the questions will help questions writers to create and improve the quality of EMQs. It allows for a formal test of the unidimensionality of the questions and thus the validity of the summed score. Given the metric calibration which follows fit to the model, it also allows for the establishment of items banks to facilitate continuity and equity in exam standards. Thus modern psychometric methods based upon the work of Rasch provide a useful approach to the calibration and analysis of EMQ undergraduate medical assessments.

## Competing interests

The author(s) declare that they have no competing interests.

## Authors' contributions

All authors have contributed to the analysis and writing of this paper and its revisions, and all have read approved the final version.

## Pre-publication history

The pre-publication history for this paper can be accessed here:


